# Build muscles and protect myelin

**DOI:** 10.1515/nipt-2024-0015

**Published:** 2024-10-16

**Authors:** Ahana Bose, Kalipada Pahan

**Affiliations:** Division of Research and Development, Jesse Brown Veterans Affairs Medical Center, Chicago, IL, USA; Department of Neurological Sciences, 2468 Rush University Medical Center, Chicago, IL, USA

**Keywords:** oligodendrocyte, myelin, HMB, multiple sclerosis

## Abstract

Multiple sclerosis (MS) is a chronic and debilitating autoimmune disease of the central nervous system (CNS) in which a CNS-driven immune response destroys myelin, leading to wide range of symptoms including numbness and tingling, vision problems, mobility impairment, etc. Oligodendrocytes are the myelinating cells in the CNS, which are generated from oligodendroglial progenitor cells (OPCs) via differentiation. However, for multiple reasons, OPCs fail to differentiate to oligodendrocytes in MS and as a result, stimulating the differentiation of OPCs to oligodendrocytes is considered beneficial for MS. The β-hydroxy β-methylbutyrate (HMB) is a widely-used muscle-building supplement in human and recently it has been shown that low-dose HMB is capable of stimulating the differentiation of cultured OPCs to oligodendrocytes for remyelination. Moreover, other causes of autoimmune demyelination are the decrease and/or suppression of Foxp3-expressing anti-autoimmune regulatory T cells (Tregs) and upregulation of autoimmune T-helper 1(Th1) and Th17 cells. Experimental autoimmune encephalomyelitis (EAE) is an animal model of MS in which the autoimmune demyelination is nicely visible. It has been reported that in EAE mice, oral HMB upregulates Tregs and decreases Th1 and Th17 responses, leading to remyelination in the CNS. Here, we analyze these newly-described features of HMB, highlighting the putative promyelinating nature of this supplement.

## Introduction

Myelin sheath is basically a lipid rich plasma membrane 1], [2], [3 for the formation of multi-layered coatings around the axon [4]. Therefore, myelin coatings keep a neuron healthy and preserve the conduction of electrical impulse through the axon. It is believed that the ideal lining by myelin is contingent on the types and proportions of lipids and proteins and myelin water fraction. Neurological dysfunction occurs when the myelin sheath is harmed and axonal padding is disturbed, resulting in the slowing down of nerve impulses along the axon. Despite intense investigations, no effective drug is available to take care of demyelination.

HMB is a popular nutritional supplement among the health and fitness community for its potential to positively modulate exercise routine and muscle growth. In addition to helping the body-building community, HMB intake is known to improve protein balance and reduce muscle wasting in cancer [5], acquired immunodeficiency syndrome (AIDS) [6], and aging [7]. Recent studies have identified neuroprotective functions of HMB in which HMB stimulates the maturation of myelin progenitor cells to myelin forming cells. In an animal model of autoimmune demyelination as well, oral administration of HMB stimulates remyelination of the CNS.

## Multiple sclerosis

Multiple sclerosis (MS), a chronic autoimmune disease with demyelination, neuronal loss, and scarring, disables around 2.8 million people worldwide usually between the ages 18–40 8], [9], [10], [11. It is known for decades that females are twice more susceptible than males to be affected by MS 12], [13], [14. As women are more prone to MS, pregnancy is a huge concern when related to MS [15]. The underlying cause of MS is not known, but the reasons are thought to be in connection with genetic and environmental factors [10, 11]. However, inflammation in the CNS plays an important role 16], [17], [18], [19], [20, which causes various motor, sensory, and visual symptoms. Cognitive impairment is likely to occur in people with MS, and that compromises the ability to do day-to-day functions. The targets of MS attacks are the myelinated axons in the CNS, and it can cause varying degrees of damage to both myelin and axons [3, 21, 22].

Diagnosis of MS can be performed with the following clinical tools: magnetic resonance imaging (MRI), evoked potentials, and monitoring of cerebrospinal fluid. MRI testing has become the primary diagnostic and monitoring device for MS [23]. Evoked potentials evaluate the efferent and afferent CNS circuits. There are three main types of MS: relapsing-remitting, secondary progressive, or primary progressive disease [24]. Most people exhibit relapsing-remitting MS, which has alternating periods of dysfunction and then having relative stability. Every relapse causes the MS to intensify. During each stability stage, the neurons rebuild their myelin sheath, but it does not go back to the normal way. Each relapse also exhibits different and new symptoms. Secondary progressive multiple sclerosis is usually what relapsing-remitting MS patients progress into years after their diagnosis [25, 26]. It is when the disease starts to become progressive without any relapses. Only a small portion of individuals are diagnosed with primary progressive multiple sclerosis phenotype [26]. This is when there is a slow progression of MS apparent from the start of the disease. These individuals do not have moments of stability as myelin-producing cells and myelin sheaths are being constantly attacked.

## Oligodendrocytes and oligodendroglial progenitor cells (OPC)

Oligodendrocytes (OLs) are a very special type of cells in the CNS that form myelin. OLs are characterized or identified by specific markers such as myelin basic protein (MBP), proteolipid protein (PLP), 2′,3′-cyclic nucleotide 3′-phosphodiesterase (CNPase), myelin oligodendrocyte glycoprotein (MOG), and galactocerebroside (GalC) 27], [28], [29. These are small cell bodies of approximately 6–8 μm in diameter that have nuclei containing a large number of chromatins. Also, cellular extensions of OLs lack fibers and are filled with cytoplasmic granules. Most OLs are capable of making about 20–60 myelinating processes with lengths between about 20 and 200 µm [30]. Myelin is a lipid membrane that wraps and insulates axons, which allows for fast-working action potentials. OLs induce many sodium channels along the axon at the nodes of Ranvier to start the saltatory conductions [31]. On the other hand, oligodendroglial progenitor cells (OPCs) are the cells that generate OLs. OPCs are also called NG2 cells or polydendrocytes because these cells express NG2 proteoglycan on the surface and have many processes [32]. While NG2 is known to stimulate the proliferation of OPC via increasing growth factor signaling [33], another cell surface ganglioside molecule A2B5 probably increases the self-renewal property of these cells [34]. Several studies have demonstrated that OPCs have an important role in remyelination, as these cells have a unique ability to proliferate and differentiate after demyelination following any insult and injury. Therefore, OPCs are key to understanding how myelin production occurs and how regeneration is able to occur in myelin when destruction happens.

## Demyelination in MS

From a pathological standpoint, MS is identified by the presence of diffuse, discrete demyelinated areas, called plaques. Demyelination involves myelin lamella stripping and using phagocytes to remove myelin fragments [35]. During demyelination, the ionic conduction through the neuron is reduced, ultimately leading to axonal loss. Therefore, demyelination is assumed as an important pathological hallmark of MS [36]. There are different mechanisms involved in the demyelination process in MS. MS involves autoreactive T cells entering the CNS to start an inflammatory cascade triggering demyelination and axonal loss [37]. CD4^+^ and CD8^+^ T cells, which are important for defense against pathogens; B cells, a type of white blood cell making antibodies; and macrophages, a type of white blood cell that surrounds and kills microorganisms, are major components of the inflammatory infiltrate into the lesions of both MS and EAE [38]. Research on demyelination in MS has been performed on animals such as mice by inducing experimental autoimmune encephalomyelitis (EAE), an autoimmune model of MS 39], [40], [41. Several studies from EAE animals have shown that nitric oxide and different proinflammatory cytokines play an important role in demyelination [22, 41, 42]. Although nitric oxide has many beneficial functions, excessive nitric oxide produced during inflammatory insults is toxic for myelin and myelin-producing OLs [43, 44].

## Role of OPC in remyelination

Myelination is a sequence of events that include the proliferation and the movement of OPCs in white matter tracts. OPCs are abundant in the CNS and during any insult or injury, OPCs are recruited to the site of injury with an ultimate goal to remyelinate demyelinated axons. However, OPCs expressing platelet-derived growth factor receptor α (PDGF-Rα), ganglioside A2B5, proteoglycan NG2, polysialic acid-neural cell adhesion molecule (PSA-NCAM), and fatty-acid-binding protein (FABP)7, are usually proliferative cells. OPCs generate OLs via maturation when OPCs acquire an elaborate morphology and lose their capacity of proliferation and migration [45, 46]. Neurobiologists have classified maturation of OPCs into different steps such as late OPC (preoligodendrocytes), immature OL (pre-myelinating) and mature OL (myelinating) [45, 46].

In MS lesions, there are few surviving OLs that can contribute to remyelination. Therefore, OPCs need to be recruited and differentiated to OLs in chronic MS lesions. If the recruitment process is too slow or inefficient, remyelination does not occur. If too slow, the OPCs reach the axons after the ‘remyelination-permissive window’ and cannot differentiate into OLs [47]. If there is not enough OPCs, the density in the lesions is below the threshold required for differentiation. Because OPC proliferation in MS lesions is little, that is mainly why there are repeated OPC differentiation [48]. The repeated demyelination/remyelination decreases the lesional OPC pool. In MS, OLs not being capable of remyelinating enough correlates with the progression of the disease.

## Muscle-building supplement HMB

Beta-hydroxy-beta-methylbutyrate (HMB) is used as a supplement for muscle-building for physically active people. HMB is found in local GNC stores as a body-building supplement. Bodybuilders consume HMB regularly to increase muscle size and muscle strength while also exercising, and to better exercise performance. HMB is known to decrease muscle protein degradation [49]. Moreover, HMB is able to increase the integrity of the myocyte cell membrane, sarcolemma [49] and activate the mTOR kinase pathway [50]. The mTOR kinase pathway is a protein kinase that controls mRNA translation efficiency. HMB improves the proliferation of muscle stem cells [49]. There is also evidence that HMB lowers the expression level of TNFα [51], a marker of inflammatory responses that occur during the recovery period. Studies have shown that HMB does have an effect on muscle strength [52]. It has also been described that HMB is more effective in untrained individuals who had periods of high physical stress. That is because people with training already have adapted to training, so the effects are reduced. Because of multiple reasons such as the increase of satellite cells and in cholesterol synthesis, there is increased tissue repair in individuals. As HMB has qualities such as resistance to fatigue and regenerative capacity [53], it is better for individuals with illnesses that cause motor problems and fatigue to use this as a supplement. HMB treatment leads to increase in fat-free mass associated with decrease in fat mass in a 12-week randomized, double-blind, placebo-controlled crossover study among 42 highly-trained combat sports athletes [54]. HMB does not exhibit any side effect even after long-term use. Therefore, HMB is considered as a safe supplement in humans.

## Maturation of OPC by HMB

Normally, OPCs should differentiate into OLs. Yet in MS, OPCs fail to differentiate into OLs [31]. For this reason, there could be therapeutic benefits for MS if there was an increase in the maturation of OPCs to OLs by any nontoxic compound/drug. HMB has been tested to study if it stimulates the maturation of OPCs into OLs. Increase in oligodendrocyte markers such as MBP, PLP, MOG, and CNPase along with subsequent reduction of NG2 and A2B5, markers of OPCs, is a reliable way to study the maturation of OPCs to OLs [27, 55], [56], [57. HMB treatment rapidly upregulates MBP and PLP and downregulates NG2 and A2B5 in OPCs, indicating differentiation into OLs [57].

**Figure 1:**
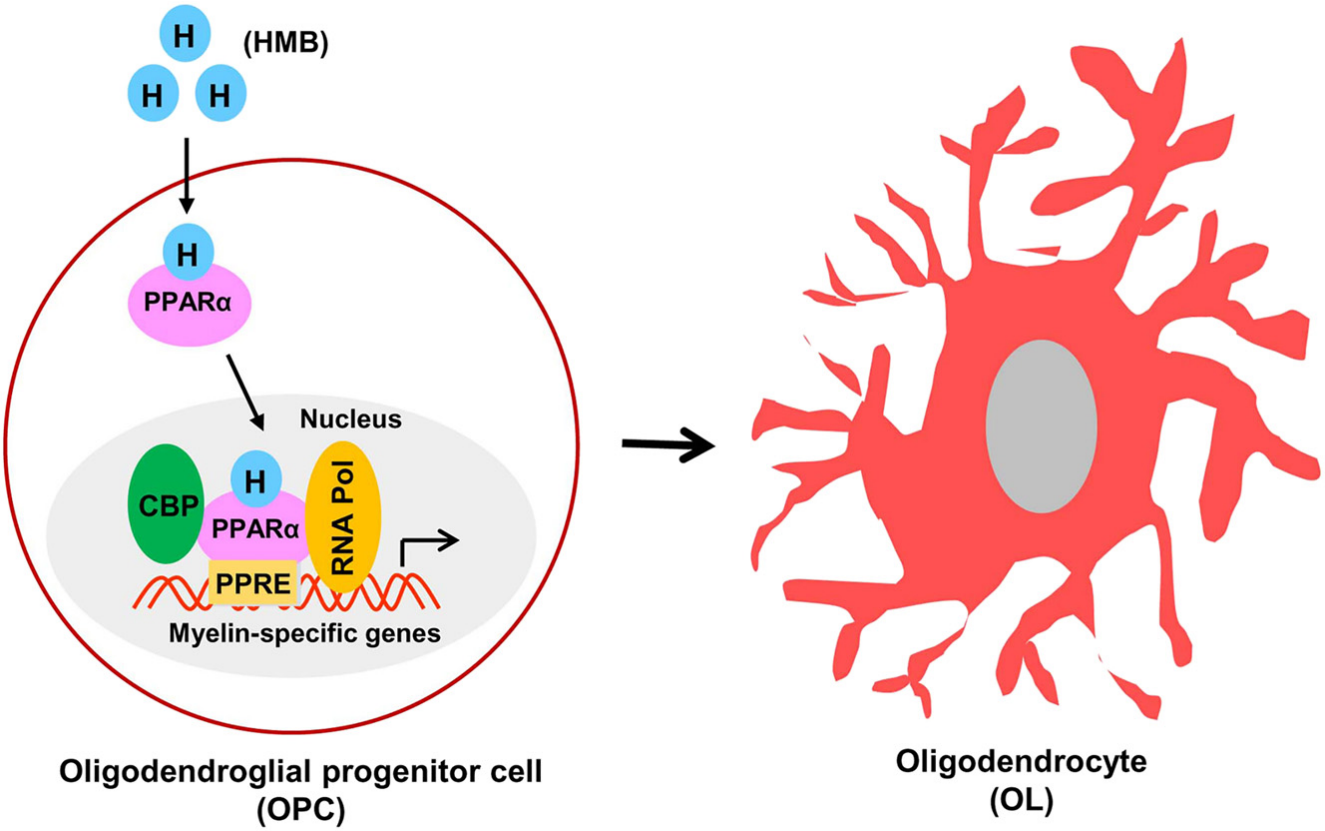
Maturation of OPC to OL by HMB. HMB stimulates the translocation of PPARα from cytoplasm to nucleus, which then binds to PPRE present in the promoter of myelin-specific genes. CBP and RNA polymerase also bind to PPRE to drive the transcription of myelin-specific proteins, ultimately leading to the maturation to OL.

While investigating underlying mechanisms, Jana et al. [57] have analyzed gene promoters of different OPC- and OL-associated markers and seen the presence of one or more peroxisome proliferator responsive element (PPRE) in the promoters of *MBP*, *PLP* and *MOG* genes, but not *NG2* and *A2B5* genes. Although the major role of peroxisome proliferator-activated receptors (PPARs), nuclear hormone receptor family of transcription factors [58, 59], is to control the metabolism of lipids [60, 61], one recent study has shown the involvement of PPARα in HMB-mediated differentiation of OPCs [57]. Interestingly, HMB binds to the ligand-binding domain of PPARα [62] and in cultured OPCs, it leads to nuclear localization of PPARα, but not PPARβ [57]. The role of PPARα is further validated by the results that PPARα agonist GW7647, but neither PPARβ agonist GW0742 nor PPARγ agonist GW1929, stimulates the maturation of OPCs into OLs [57]. Similarly, PPARα antagonist GW6471, but neither PPARβ antagonist GSK0660 nor PPARγ antagonist GW9662, inhibits HMB-induced maturation of OPCs. Accordingly, HMB remains unable to stimulate the differentiation of OPCs isolated from PPARα^−/−^ mice into OLs [57]. It has been also shown that HMB treatment causes the recruitment of PPARα, but neither PPARβ nor PPARγ, in cultured OPCs. Although there are two prominent histone acetyl transferases (p300 and CREB-binding protein or CBP) 63], [64], [65, HMB retains CBP, but not p300, to the PPRE of myelin gene promoter in OPCs [57]. It has been also delineated that a complex of PPARα, CBP and RNA polymerase II is involved in the transcription of myelin genes in HMB-treated OPCs (Figure 1). Therefore, this newly-described promyelinating function of HMB is dependent on the transcriptional activity of PPARα (Figure 1).

## Stimulation of remyelination in an animal model of MS by HMB

As demyelination in the CNS is a major feature in MS [66], and EAE serves as an animal model of MS [17, 67, 68], studies have also tested the effect of HMB on demyelination and the disease process of EAE. In MS as well as EAE, spinal cord is affected the most and accordingly, myelin staining by Luxol fast blue identifies widespread demyelination zones in the white matter of EAE mice [69, 70]. However, oral HMB markedly improves the myelin level in spinal cord of EAE mice [71]. The mRNA levels of myelin markers such as MBP and PLP is also normalized in the spinal cord of EAE mice by HMB treatment [71]. The ultimate goal of neuroprotective therapies of MS is to decrease functional impairments. Accordingly, oral HMB has been shown to suppresses clinical symptoms of EAE in mice [71]. Since the autoimmune component plays an important role in the pathogenesis of EAE in mice, the effect of HMB has been also tested on immunomodulatory subtype of T lymphocytes in EAE. Regulatory T cells (Tregs) are viewed as a necessary immunomodulatory subtype of T lymphocytes, which become defective during the autoimmune attacks 72], [73], [74. It is also believed that due to downregulation of Tregs, there is upregulation of effector Th1 and Th17 cells in EAE and MS 75], [76], [77. Interestingly, oral HMB also stimulates the anti-autoimmune Treg response and attenuates the autoimmune Th1 and Th17 responses in EAE mice [71].

## Conclusions

**Figure 2:**
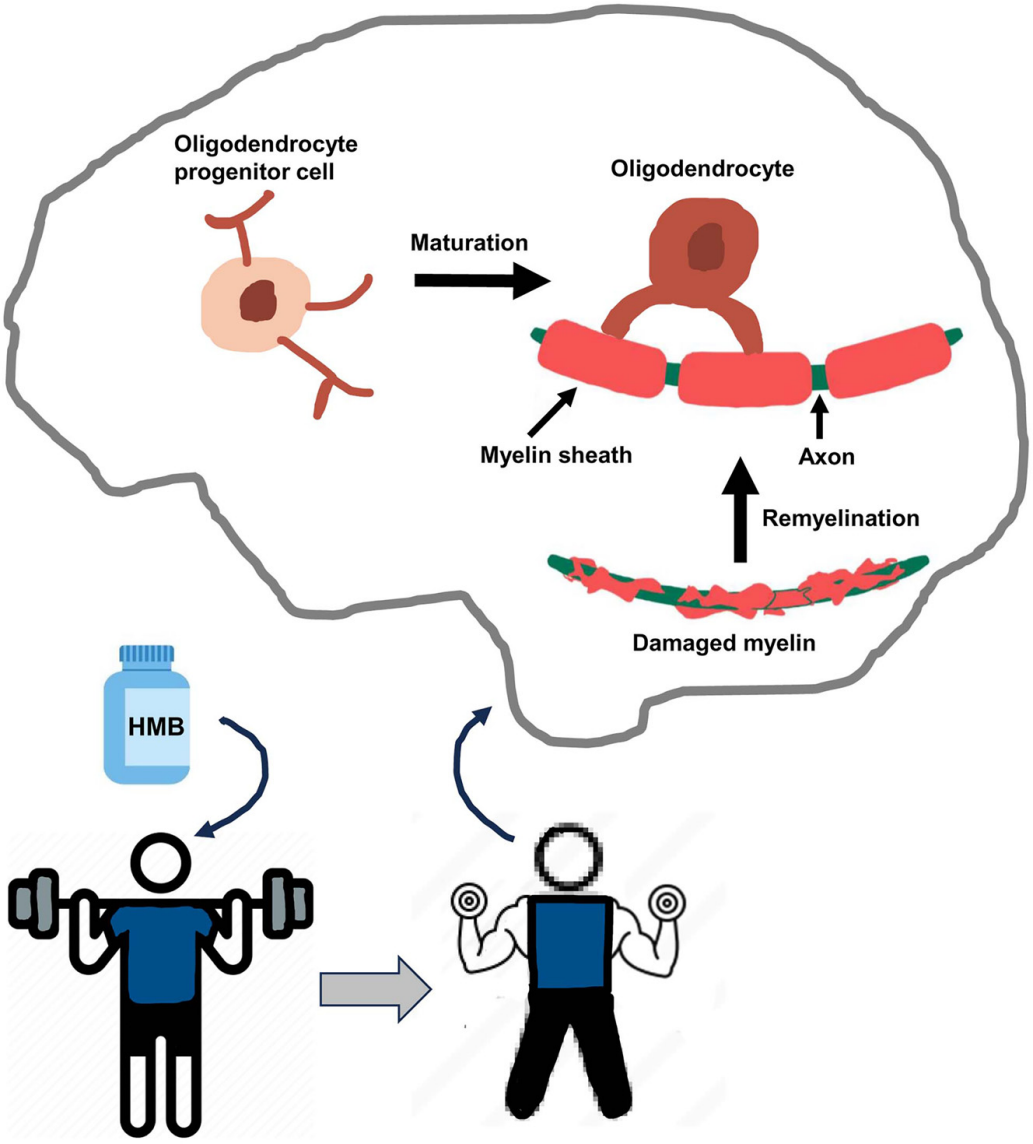
Schematic presentation of remyelination by body-building supplement HMB. While HMB and exercise help in body building, it also leads to the maturation of OPC to OL in the CNS, resulting in remyelination.

Currently, there is no effective treatment for remyelination and as a result, available treatments only control demyelination symptoms of MS to a certain extent to provide some relief from the pain and suffering due to this disease. Furthermore, FDA has not yet approved any medicine for treating primary progressive MS. HMB is a popular nutritional supplement among fitness lovers for its efficacy in supporting exercise routine and muscle growth. Additionally, HMB is reported to improve protein balance and reduce muscle wasting in patients with acquired immunodeficiency syndrome (AIDS) [6], cancer [5], and aging [7]. As compared with the placebo, HMB supplementation has been also reported to reduce total cholesterol (5.8 %, p<0.03), decrease LDL cholesterol (7.3 %, p<0.01) and lower systolic blood pressure (4.4 mm Hg, p<0.05) [78]. Therefore, it is stimulating to identify the likelihood that low dose of HMB may have remyelinating efficacy in MS (Figure 2). *First,* HMB stimulates the differentiation of cultured OPCs to OLs. *Second,* oral HMB increases myelination of demyelinated spinal cord in EAE mice. *Third,* HMB also reduces clinical symptoms of autoimmune demyelination in EAE mice. Moreover, HMB treatment suppresses autoimmune Th1 and Th17 signaling and augments/restores anti-autoimmune Treg signaling in EAE mice. If cultured OPC results and mouse EAE results are translated, oral HMB may stimulate remyelination via upregulation of OPC maturation, suppression of Th1/Th17 signaling, and restoration of Treg signaling. Since HMB has demonstrated nice safety and tolerability records in different clinical trials, this muscle-building friend may be considered to be repurposed for remyelination in MS (Figure 2). Although there is no evidence of direct crosstalk between muscles and myelin, exercise is known to support muscle strengthening and growth. Recently, it has been described that exercise stimulates the activation of PPARα in the brain [79, 80]. Since PPARα is involved in the maturation of OPCs via direct transcriptional upregulation of myelin-specific genes [57], muscle strengthening via exercise should be helpful for the maturation of OPCs and hence myelination (Figure 2).
